# The Multifaced Role of STAT3 in Cancer and Its Implication for Anticancer Therapy

**DOI:** 10.3390/ijms22020603

**Published:** 2021-01-09

**Authors:** Manlio Tolomeo, Antonio Cascio

**Affiliations:** Department of Health Promotion Sciences, Maternal and Infant Care, Internal Medicine and Medical Specialties, University of Palermo, via del Vespro 129, 90127 Palermo, Italy; antonio.cascio03@unipa.it

**Keywords:** STAT3, cancer, tumor promoter, tumor suppressor

## Abstract

Signal transducer and activator of transcription (STAT) 3 is one of the most complex regulators of transcription. Constitutive activation of STAT3 has been reported in many types of tumors and depends on mechanisms such as hyperactivation of receptors for pro-oncogenic cytokines and growth factors, loss of negative regulation, and excessive cytokine stimulation. In contrast, somatic STAT3 mutations are less frequent in cancer. Several oncogenic targets of STAT3 have been recently identified such as c-myc, c-Jun, PLK-1, Pim1/2, Bcl-2, VEGF, bFGF, and Cten, and inhibitors of STAT3 have been developed for cancer prevention and treatment. However, despite the oncogenic role of STAT3 having been widely demonstrated, an increasing amount of data indicate that STAT3 functions are multifaced and not easy to classify. In fact, the specific cellular role of STAT3 seems to be determined by the integration of multiple signals, by the oncogenic environment, and by the alternative splicing into two distinct isoforms, STAT3α and STAT3β. On the basis of these different conditions, STAT3 can act both as a potent tumor promoter or tumor suppressor factor. This implies that the therapies based on STAT3 modulators should be performed considering the pleiotropic functions of this transcription factor and tailored to the specific tumor type.

## 1. Introduction

Signal transducer and activator of transcription 3 (STAT3) is a multifunctional transcription factor involved in multiple biological processes. It was identified in 1993 by Wegenka et al. [1]. These authors observed that interleukin-6 (IL-6) rapidly induced the activation of a DNA-binding factor, termed acute-phase response factor (APRF), in rat liver and in human hepatoma cells. In 1994, Zhong et al. [2] described a 92 kD mouse protein from the signal transducer and activator of transcription (STAT) family that was activated as a DNA-binding protein by IL-6 and by epidermal growth factor (EGF). The partial amino acid sequence obtained from purified rat APRF and the use of a specific antiserum demonstrated that APRF was related to STAT3 [3].

STAT3 belongs to the STAT family of cytoplasmic transcription factors that mediate signal transduction from the plasma membrane to the nucleus in various cellular activities [4].

There are seven STAT proteins: STAT 1, 2, 3, 4, 5A, 5B, and 6 encoded by genes clustered on different chromosomes. Each of them consists of six regions: (1) a helical N-terminal domain (ND) for protein–protein interactions between adjacent STAT dimers on DNA; (2) a coiled-coil (CC) domain for interactions with regulatory proteins that positively or negatively modulate the transcriptional activity; (3) a DNA-binding domain (DBD) for the recognition of specific DNA-sequences of target genes; (4) a helical linker (LK) domain involved in nuclear export and DNA binding; (5) an Src homology 2 (SH2) domain for receptor binding and dimerization; (6) a C-terminal transactivation domain (TAD) that contains specific residues that are phosphorylated upon transcriptional activation [5,6].

STATs are activated in the cytoplasm by Janus kinases (JAKs), a family composed of four different intracellular non-receptor tyrosine kinases, that transduce cytokine-mediated signals [7,8]. JAKs are associated with two different types of receptors (type I and type II) that do not possess any catalytic activity but, after binding with the specific ligand, allow the auto-phosphorylation of JAK, which, in turn, phosphorylates and activates STAT proteins [9].

STATs recognize DNA motifs with a consensus sequence of 5′-TTCN3/4GAA-3′ that are located in promoter and enhancer regions and in the first introns of target genes [10].

STAT3 is one of the most complex transcription regulators and is involved in many biological functions such as cell proliferation, maturation, and survival. STAT3 is activated by the entire family of IL-6-type cytokines comprised of IL-6, IL-11, IL-22, IL-27, IL-31, oncostatin M (OSM), cardiotrophin 1 (CT-1), ciliary neurotrophic factor (CNTF), cardiotrophin-like cytokine factor 1 (CLCF1), and leukemia inhibitory factor (LIF) [11,12]. These cytokines are involved in embryonic development, immunologic activity, inflammation, hematopoiesis, cardiovascular physiology, liver, and neuronal regeneration.

A main role in the IL-6 cytokine family/STAT3 axis is played by a transmembrane—cytokine receptor associated—protein of 130 kD called gp130. In fact, this protein mediates, as a common signal transducer, the pleiotropic but overlapping functions of the IL-6 cytokine family. When a member of the IL-6-type cytokine family binds to its receptor, it induces the recruitment of gp130 and the formation of a complex of three proteins (IL-6-type cytokine/IL-6 member receptor/gp130). After complexing, gp130 is phosphorylated on tyrosine residues. The phosphorylation leads to the association with JAK, which, in turn, activates STAT3, which leads to the activation of downstream genes. Animals lacking gp130 are not viable, indicating the importance of this receptor associated protein [13].

While gp130 expression is relatively ubiquitous in a wide variety of tissues and organs, receptors of the IL6-cytokine family are more restricted and cell-type specific. These receptors include IL-6R (IL-6 receptor), IL-11R (IL-11 receptor), IL-27Rα (IL-27 receptor alpha), OSMR (OSM receptor), LIFR (LIF receptor), and CNTFRα (CNTF receptor alpha).

Many studies have demonstrated the critical role of aberrant STAT3 in malignant transformation, and several STAT3 oncogenic targets have been identified. However, recent evidence has shown that STAT3 can have opposite functions in cancer, and based on different conditions, STAT3 can act as both a potent tumor promoter and a tumor suppressor factor. This appears to depend on several factors such as the integration of multiple signals, the oncogenic environment, and the alternative splicing into different isoforms.

## 2. STAT3 and Tumorigenesis

STAT3 is currently considered an oncogene, and aberrant regulation of STAT3 has been reported in nearly 70% of cancers [14,15]. Aberrant activation of STAT3 in cancer cells causes the continuous transcription of cell growth factors and anti-apoptotic molecules that play a crucial role in maintaining cell growth and survival [16]. Moreover, STAT3 confers tumor great malignancy by promoting tumor invasion, migration, metastasis, and angiogenesis. This has led many researchers to develop STAT3 inhibitors for the treatment of different malignancies.

The first study demonstrating the involvement of STAT3 in cancer was published in 1995 by Yu et al. They showed that oncoprotein Src can activate the STAT3 signaling pathway, raising the possibility that this transcription factor contributes to oncogenesis by Src [17]. In 1996, Cao et al. reported that in all v-Src-transformed cell lines examined, STAT3 was constitutively activated, and phosphorylation of tyrosine on STAT3 was enhanced by the induction of v-Src expression [18]. They also showed that Src was associated with the presence of a constitutively activated STAT3 in vivo [18]. Although Src activation of STAT3 is the most well-characterized model, there was evidence that STAT3 can be activated by other members of the Src family kinases [19] and by other oncogenic tyrosine kinases, such as v-Ros, v-Fps, Etk/BMX, and v-Abl [20,21,22,23,24,25], or by viral proteins that directly or indirectly activate tyrosine kinase pathways, including human T lymphotropic virus (HTLV)1, polyomavirus middle T antigen, Epstein–Barr virus (EBV), and herpes virus saimiri [26,27,28,29,30,31].

It is noteworthy that Bromberg et al. reported that the substitution of two cysteine residues within the C-terminal loop of the SH2 domain of STAT3 produces a molecule that dimerizes spontaneously, binds to DNA, and activates transcription, causing cellular transformation, thus demonstrating that the activated STAT3 molecule by itself can mediate cellular transformation [32].

Hyperactivation of STAT3 has been reported in many types of tumors and can depend on the following mechanisms: (i) hyperactivation of receptors for pro-oncogenic cytokines and growth factors; (ii) elevated activity of cytoplasmic non-receptor tyrosine kinases, such as Src, Janus kinases (JAKs), and Abelson (Abl) kinase; (iii) loss of negative STAT3 regulation; (iv) excessive stimulation by cytokines such as IL-6 or EGF. These mechanisms can induce uncontrolled cell growth, malignant cell transformation, angiogenesis, metastasis, invasion, and immune escape. For example, the growth and survival of human myeloma cells is dependent on an IL-6 autocrine loop that induces a constitutive activation of STAT3 [33]. Likewise, IL-6 is an autocrine growth factor for human prostate cancer cells, and the effects of IL-6 on prostate cancer cell growth are mediated through the Jak/STAT3 signaling pathway [34]. Elevated epidermal growth factor receptor (EGFR)-mediated signaling or Src and Jak kinases’ activities have been observed in breast cancer, prostate cancer, non-small cell lung cancer (NSCLC), melanoma, pancreatic cancer, and head and neck squamous carcinoma (HNSCC) cells [23,35,36,37,38,39,40].

In contrast, somatic STAT3 mutations are less frequent in cancer. For example, sixty percent of inflammatory hepatocellular adenomas have IL-6 signal transducer mutations that cause hyperactivation of IL-6/STAT3 signaling, while 12% of these tumors lacking IL-6 signal transducer mutations have somatic STAT3 mutations [41].

However, some types of cancers frequently have STAT3 mutations. For example, STAT3 mutations were found in 40% of patients with large granular lymphocytic leukemia. In this leukemia, all mutations were located in exon 21, encoding the Src homology 2 (SH2) domain, which mediates the dimerization and activation of STAT3 [42].

**Figure 1:**
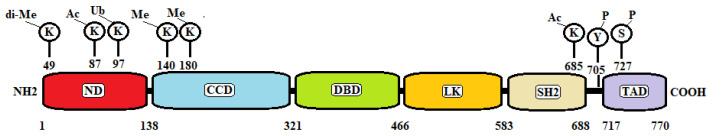
Structure of signal transducer and activator of transcription 3 (STAT3). Functional domains: ND, NH2-terminal domain; CCD, coiled-coil domain; DBD, DNA-binding domain; LK, linker domain; SH2, Src homology 2 domain; TAD, transactivation domain. STAT3 activation is dependent on the phosphorylation (P) of a tyrosine residue Y705, which is located between the SH2 domain and TAD. Phosphorylation on serine (S) 727 is required for maximal transcriptional activity. After cytokine treatment, STAT3 is acetylated (Ac) on lysine (K) K87 and 685. Other post-translational modifications are di-methylation (di-Me) of K49, monoubiquitination of K97, and methylation (Me) of K140 and K180.

STAT3 activation in normal and tumor cells is dependent on the phosphorylation of a tyrosine residue (Y705), which is located between the SH2 domain and TAD (Figure 1). Phosphorylation of Y705 is crucial for STAT3 dimerization, nuclear translocation, and DNA binding. A second phosphorylation site is located on serine 727 (S727) in the C-terminal domain (Figure 1). Phosphorylation on S727 is required for maximal transcriptional activity. However, its functions are probably more complex since the S727 site is phosphorylated in response to various cellular stresses and through the interaction with transcriptional coactivators such as SRC, Cdk9, or CBP in the absence of Y705 phosphorylation [43,44]. In chronic lymphocytic leukemia, STAT3 is constitutively phosphorylated on S727 and not on Y705, and dephosphorylation of inducible tyrosine pSTAT3 does not affect STAT3-DNA binding [45]. It has also been reported that Ras-mediated transformation is significantly reduced when STAT3 is mutated on its S727 residue. Malignant transformation by activated Ras is impaired without STAT3, in spite of the inability of Ras to drive STAT3 tyrosine phosphorylation or nuclear translocation. The cooperation between Ras and STAT3 requires the serine phosphorylation site at the C-terminus. The mutation of S727 or deletion of the C-terminal domain abrogated cooperation between STAT3 and Ras [46]. Some observations suggest that phosphorylation on S727 could be connected to the genetic instability and DNA damage that occur early in tumor cells [47].

After cytokine treatment, STAT3 is also acetylated on a single lysine (K) residue, K685 (Figure 1). Acetylation of K685 is critical for STAT3 to form stable dimers required for cytokine-stimulated DNA binding and transcriptional regulation. This site of acetylation also plays an important role in cancer growth. In fact, lysine acetylation of STAT3 is elevated in tumors such as melanoma and colorectal cancer. Genetically altering STAT3 at K685 reduces tumor growth, which is accompanied by demethylation and reactivation of several tumor-suppressor genes [48].

Other post-translational STAT3 modifications are p300-mediated acetylation of K87 at the STAT3 NH2 terminus, which is required for IL-6-induced target gene activation [49], dimethylation of K49 at the NH2 terminus, which is crucial for the expression of many IL-6-dependent genes [50], monoubiquitination at K97, which plays a role in STAT3 antiapoptotic gene expression [51], methylation of K140, which is a negative regulatory event, because its blockade greatly increases the amount of activated STAT3 [52], and methylation of K180, which leads to enhanced STAT3 activity [53] (Figure 1).

STAT3 regulates multiple biological functions in the initiation of malignant transformation, and it is the convergence point of several major oncogenic signaling pathways. Moreover, STAT3 modulates host immunity against tumor cells leading to tumor-induced immunosuppression. This depends on the ability of STAT3 to suppress anticancer immune cells and to activate cancer promoting immune cells. Therefore, therapies based on STAT3 modulators can not only directly inhibit the growth of tumors, but also enhance antitumor immunity.

## 3. Role of STAT3 in Oncogenic Transformation

**Figure 2:**
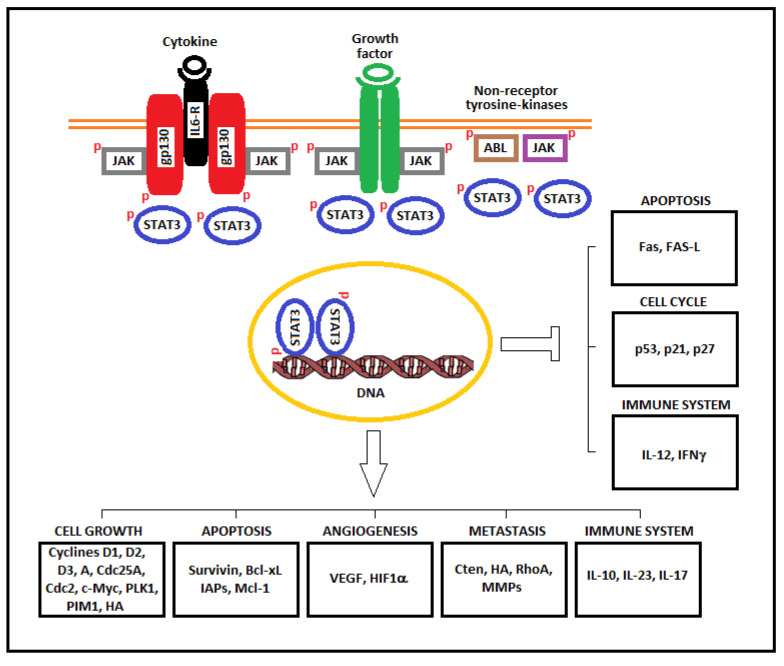
STAT3 upregulation and downregulation of factors involved in cell growth, apoptosis, angiogenesis, invasion, metastasis, and the immune system. STAT3 is activated through the interaction of cytokines and growth factors. Non-receptor tyrosine kinases have intrinsic kinase activity, whereas the receptors of ligands have associated JAK, which, when phosphorylated, acts as a platform for un-phosphorylated STAT3 to become activated. Phosphorylated STAT3 dimers translocate to the nucleus where they upregulate and downregulate a variety of genes that can contribute to tumorigenesis.

Activated STAT3 can upregulate the mRNA levels of many genes involved in cell growth and apoptosis such as cyclins D1, D2, D3, A, and B, Cdc25A, Cdc2, c-Myc, PLK1, Pim-1/2, Cten, survivin, Bcl-xL, IAPs, and Mcl-1. These upregulated genes cooperate in inducing the oncogenic transformation of cells. In addition, STAT3 downregulate genes encoding cell cycle checkpoint proteins such as p21, p27, and p53. Other genes, such as c-Jun, c-Fos, and FGFR, interact with STAT3 in inducing cancer transformation (Figure 2).

STAT3 plays a key role in the G1 to S phase cell cycle transition through the upregulation of cyclins D1, D2, D3, A, and Cdc25A and the concomitant downregulation of p21 and p27. Moreover, STAT3 participates in modulating the G2–M phase checkpoint by regulating gene expressions of cyclin B1 and Cdc2 via E2F [54]. Cyclin D1 mRNA levels are increased in primary rat-, mouse-, and human-derived cell lines expressing either the oncogenic variant of STAT3 (STAT3-C) or vSrc, which constitutively phosphorylates STAT3 [55]. Cyclin D1 mediates the progression of cells from the G1 to S phase of the cell cycle by phosphorylating the retinoblastoma protein (Rb). Phosphorylation of the Rb protein releases the E2F transcription factor, leading to transcription and S phase entry. Overexpression of cyclin D1 is found in many cancers and is sufficient to mediate mammary tumorigenesis [56,57]. Furthermore, cyclin D1-deficient animals are resistant to Ras- and Neu-mediated skin and breast tumorigenesis [58,59].

c-Myc is another important downstream effector of STAT3 signaling involved in cell growth and transformation. Disruption of STAT3 signaling by using dominant-negative STAT3β protein in NIH 3T3 fibroblasts suppresses c-Myc expression. Moreover, fibroblasts with c-Myc gene knockout are refractory to transformation by v-Src, and disruption of STAT3 signaling in normal cells inhibits PDGF-induced mitogenesis in a manner that is reversed by ectopic c-Myc expression [60].

Zhang et al. reported that there is an interaction between a region within c-Jun and specific sites within STAT3 [61]. c-Jun was the first oncogenic transcription factor discovered [62]. It is required for progression through the G1 phase of the cell cycle and protects cells from UV-induced apoptosis. Mutations in the contact region of STAT3 both reduce c-Jun–STAT3 protein interaction and disrupt the cooperation between these two proteins, which is required for maximal IL-6-dependent gene activation driven by the α2-macroglobulin enhancer [61,63]. c-Fos encodes a 62 kDa protein, which forms a heterodimer with c-Jun, resulting in the formation of the AP-1 (activator protein-1) complex, which binds DNA at AP-1 specific sites. It plays an important role in many cellular functions and has been found to be overexpressed in a variety of cancers. Overexpression of c-Jun and c-Fos strongly enhances STAT3-driven gene transactivation [64].

Two other factors implicated in cell growth and apoptosis and modulated by STAT3 are PLK-1 and Pim 1 and 2. Serine/threonine-protein kinase PLK1, also known as polo-like kinase 1 (PLK-1), is an enzyme that in humans promotes in cells the G2/M transition. It is considered a proto-oncogene, whose overexpression is often observed in tumor cells. The oncogenic properties of PLK1 are believed to be due to its role in driving cell cycle progression. In addition, PLK1 can inhibit the transactivation and pro-apoptotic functions of p53. STAT3 and PLK1 control each other’s transcription in a positive feedback loop that contributes to the development of cancer such as esophageal squamous cell carcinoma [65]. The PIM family of serine/threonine kinases possess weak oncogenic abilities, but enhance other genes or chemical carcinogens to induce tumors. Pim-1 and Pim-2 are targets for STAT3 signal and the expression of a kinase-defective Pim-1 mutant attenuated STAT3-mediated cell proliferation. Furthermore, constitutive expression of Pim-1 together with c-Myc fully compensated for the loss of the STAT3-mediated cell cycle progression, antiapoptosis, and Bcl-2 expression [66].

STAT3 activation upregulates factors implicated in cell motility and malignant transformation such as COOH terminal tensin-like (Cten). Tensin is a signaling molecule that binds to actin filaments and localizes to focal adhesions. Cten is an atypical tensin family member lacking the actin-binding domain that promotes colon cancer tumorigenicity and cell motility [67,68]. In addition, its overexpression correlates with increasing tumor stage in thymomas, lung tumors, and gastric tumors [69,70,71].

A novel pathway that contributes to tumor growth, involving fibroblast growth factor receptors (FGFRs), STAT3, and hyaluronan (HA) synthesis, has been recently described by Bohrer et al. [72]. FGFR is a transmembrane receptor tyrosine kinase that is activated by FGF. Aberrant expression of these receptors was observed in different cancers such as cholangiocarcinoma, breast cancer, and prostate cancer [73]. Activation of FGFR induces the production of IL-6 family members, which activate the STAT3 pathway. FGFR-induced STAT3 activation contributes to the synthesis of hyaluronan (HA), a glycosaminoglycan, which, interacting with cancer, promotes proliferation and migration.

Finally, STAT3 inhibits apoptosis by modulating five important factors implicated in apoptosis regulation: survivin, Bcl-xL, IAPs, Mcl-1, and p53.

Survivin is an inhibitor of apoptosis. It acts by inhibiting caspase activation, thereby leading to the negative regulation of apoptosis. Survivin protein is overexpressed in most human tumors and fetal tissues. Survivin gene has been identified as a STAT3-regulated gene in breast cancer cells [74]. Direct inhibition of activated STAT3 signaling with antisense oligonucleotides inhibits survivin expression. Moreover, the block of constitutively active STAT3 or survivin expression in breast cancer cells by antisense oligonucleotides induces apoptosis. Both constitutive STAT3 activation and elevated survivin expression occur concurrently in high-risk breast tumors that are resistant to chemotherapy with docetaxel and doxorubicin [74].

Bcl-xL is another important anti-apoptotic factor, and its expression level is upregulated by STAT3 other than by other factors such as NF-κB and tumor necrosis factor-alpha (TNF-α). Inhibition of STAT3 leads to a decrease in the expression of Bcl-xL [75]. In a study designed to investigate the potential use of RNA interference (RNAi) to block STAT3 expression and activation on human breast cancer cells, Kunigal et al. showed that the knockdown of STAT3 expression by siRNA reduced the expression of Bcl-xL and survivin. Of interest, knockdown of STAT3 caused also a Fas and Fas-L upregulation, induction of apoptosis, and tumor suppression. This study confirms that STAT3 contributes to the inhibition of apoptosis in tumor cells by acting on the expression of anti-apoptotic factors and downregulating the extrinsic apoptotic pathway [76].

Activation of STAT3 increases the transcription and expression of the IAP (inhibitor of apoptosis proteins) family proteins and Mcl-1 [77]. IAPs are a family of functionally and structurally related proteins that act as endogenous inhibitors of programmed cell death. They play a role in oncogenesis by suppressing apoptosis. IAPs inhibitors, such as LCL161, promote cancer cell death by antagonizing IAPs. Mcl-1 (myeloid cell leukemia-1) is a protein that belongs to the Bcl-2 family. Amplification and overexpression of Mcl-1 have been reported in various human tumors, including hematological malignancies and solid tumors [78].

STAT3 is able to modulate the expression of p53, one of the most important oncosuppressor proteins, which plays a crucial role in many tumors. In fact, the p53 gene is frequently mutated in cancer, but p53 mutations generally occur at a late stage in tumor development. In contrast, in the early stage, many clinically detectable cancers have only reduced p53 expression. STAT3 seems to play an important role in reducing p53 transcription by binding to the p53 promoter. Site-specific mutation of an STAT3 DNA-binding site in the p53 promoter partially abrogates STAT3-induced p53 inhibition, and the blocking of STAT3 in cancer cells upregulates expression of p53, leading to p53-mediated tumor cell apoptosis [79].

All these data indicate the complex role of STAT3 in modulating cell growth, apoptosis, and tumorigenesis. However, many tumors express pro-survival and growth genes in a non-STAT3-dependent manner. In other tumors, STAT3 activation alone appears insufficient to induce tumorigenesis, but acts by conferring greater malignancy to neoplastic cells. This was demonstrated in two different studies. In a mouse model of tumorigenesis study, STAT3 did not alter mammary tumor initiation but affected metastatic progression. In fact, only a few mice bearing STAT3-null tumors showed lung metastasis and lung lesions compared with the wild-type cohort. Tumors from STAT3-null mice were less vascularized than tumors from the wild-type animals and showed a significant reduction of the proangiogenic factor, VEGF. Moreover, a gene expression analysis showed that STAT3-null mice expressed lower levels of both cebpd and osmr, two genes that are induced by STAT3. These genes code for C/EBPδ and the oncostatin M receptor, two factors that are known to potentiate the acute-phase response of inflammation [80].

Similar results were obtained by Babieri et al. in mice expressing the activated rat ErbB-2 (neu) but lacking STAT3 in the mammary epithelium. STAT3 was apparently not required for neu-driven mammary tumorigenesis. However, STAT3 silencing in a neu-overexpressing tumor-derived cell line completely abolished both neu-driven anchorage-independent growth and lung metastasis [81].

These data indicate that STAT3 confers tumor great malignancy by promoting tumor invasion, migration, metastasis, and angiogenesis. Moreover, it plays an important role in cancer immune escape.

## 4. Invasion, Metastasis, and Angiogenesis

The formation of metastases depends on the ability of cancer cells to degrade the basal membrane and extracellular matrix and to move within. STAT3 regulates both of these two processes. RhoA-Rho-associated protein kinase (ROCK) is a kinase belonging to the AGC family of serine-threonine kinases. It is involved mainly in regulating the shape and movement of cells by acting on the cytoskeleton. The ROCK-myosin axis is the most well-known mechanism of cell contractility and is the major signaling pathway that induces amoeboid movement [82,83]. Amoeboid movement has been described for many cells including cancer cells [84]. STAT3 coordinates ameboid movement through activating RhoA, and inhibition of JAK/STAT3 activity suppresses the migration of malignant cells [85]. Invasion of the extracellular matrix is a key step in tumor metastasis formation, and several pieces of evidence indicate that STAT3 plays a crucial role also in this process by regulating the matrix metalloproteinases (MMPs), a family of zinc-dependent endoproteinases whose enzymatic activity is directed against components of the extracellular matrix such as collagens, laminins, and proteoglycans. STAT3 protein directly binds to the promoter of MMP genes, upregulating their expression and favoring the invasiveness of cancer cells [86,87,88]. On the contrary, STAT3 knockdown reduces MMP expression and cancer cell invasiveness in nude mice [89].

After degradation of the basement membrane, tumor cells enter the circulatory or lymphatic system, forming metastasis in lymph nodes and in distant organs. STAT3 activation has an important role in protecting metastatic cells from the immune system by modulating the secretion of various inflammatory factors such as IL6 and TNFα [90] and reducing the activity of NK cells [91].

Another essential step for tumor growth and metastasis formation is angiogenesis, which is mediated by the vascular endothelial growth factor (VEGF) [92]. STAT3 is a direct transcriptional activator of the VEGF gene [93]. Moreover, STAT3 induces the expression of hypoxia-inducible factor-1α (HIF1α), which is another key mediator of angiogenesis [94]. Both STAT3 and HIF1α bind simultaneously to the VEGF promoter, leading to its maximum transcriptional activation and angiogenesis [95].

## 5. Immune Escape

The immune system can suppress or promote cancer depending on the type of immune cells. Innate immune cells (such as macrophages, natural killer, and dendritic cells) and cells from the adaptive immune system (Th1 cells) can destroy tumor cells when activated [96]. In contrast, tumor-associated macrophages and MDSCs promote cancer through their ability to secrete angiogenetic, metastatic, and growth factors [97,98] or by suppressing anti-tumor immunity [99]. Evidence indicates that STAT3 is involved in the immune escape of tumor cells through the suppression of anticancer immune cells and activation of cancer promoting immune cells.

T helper cells (Th cells), also known as CD4+ cells, play a central role in cancer immune response by activating antigen-specific effector cells and recruiting cells of the innate immune system such as macrophages and mast cells. Once activated, Th cells proliferate rapidly and secrete cytokines to regulate immune response [97].

Th cells are activated and develop into effector T cells by two costimulatory signals from antigen presenting cells (APCs). The first costimulatory signal is the stimulation with antigens presented by major histocompatibility complex class-II (MHC-II) molecules, expressed on the surface of APCs. The second costimulatory signal is mediated by the linkage between CD80 or CD86 on the APCs and CD28 on the T cells.

Activated STAT3 in cancer cells induces the release of factors that block APCs maturation/activation and inhibits the generation of antigen-specific T cells, causing immune tolerance [91]. Furthermore, STAT3 activation in APCs determines immune tolerance by preventing the activation of T cells. The tolerance of T cells can be reversed if activation of STAT3 signaling is blocked in APCs [100].

Naive CD4 + T cells that develop into effector T cells differentiate into five major subtypes of cells known as Th1, Th2, Threg, Th17, and Th9.

Th1 cell differentiation is induced in the presence of IL-12. Th1 cells produce the proinflammatory cytokine IFN-γ and are associated with a good prognosis in several cancer types. They are primarily responsible for activating and regulating the development and persistence of cytotoxic T cells (CTLs). In addition, Th1 cells activate APC. Stat1 is the key transcription factor for the IFN-γ-mediated gene [101], and NF-kB is involved in the activation of IL-12 transcription [102]. STAT3 activation inhibits anti-tumor immune response by antagonizing the NF-κB- and STAT1-mediated expression of anti-tumor Th1 cytokines such as IL-12 and IFNγ [103,104]. The ablation of the STAT3 gene in natural killer cells and macrophages in mice increases the expression of Th1 cytokines, leading to STAT1 upregulation and increased anti-tumor immune responses [105].

Th2 and Th17 cells are two other major T cell subsets found within the tumor microenvironment. Th2 cells produce anti-inflammatory cytokines, whereas Th17 cells produce mainly proinflammatory cytokines including IL-17 and IL-22. These two cell types appear to be overall pro-tumoral.

The Th2 anti-inflammatory cytokine IL-10 is involved in cancer development and is transcriptionally upregulated by STAT3 in tumors. IL-6 promotes STAT3 recruitment in both colon cancer cells and T cells, which, in turn, upregulates IL-10 secretion [106]. IL-10 downregulates the expression of Th1 cytokines and MHC class II antigens and blocks NF-κB activity.

IL-6, transforming growth factor-β (TGFβ), and IL-23 are involved in the activation of Th17 immune response. In contrast to the tumor suppressor role of Th1 immune response, Th17 response and IL-23 play a cancer-promoting role. STAT3 is involved in Th17 development because it acts both as the major IL-6R-dependent transcription factor in T cells and as a transcriptional activator of Il-23a that encodes part of IL-23 [107]. Furthermore, the STAT3- and Th17-dependent pathways are involved in inflammation-induced cancer by a common human commensal bacterium [108].

Treg cells are characterized by the production of major suppressive cytokines, including IL-10 and TGF-β. The presence of Treg cells in the tumor correlates mainly with a bad prognosis. Treg cells secrete IL-10 and TGF-β. TGF-β in turn induces the expression of Forkhead box P3 (FOXP3), a mediator of Treg cells that converts naive CD4+ T cells into CD4+CD25+ FOXP3+ Treg cells [109,110]. STAT3 was demonstrated to transcriptionally upregulate the expression of FOXP3 in CD4+CD25+ Treg cells by binding to the promoter of FOXP3 [111].

Finally, STAT3 signaling is required for the immunosuppressive and tumor-promoting effects of myeloid-derived suppressor cells (MDSCs), tumor-associated macrophages [112], and in expansion of T regulatory cells, which promote tumor progression by inhibiting anti-tumor immune responses mediated by Th1-type CD4+ T cells and CD8+ T cells [113,114,115,116].

## 6. The Dual Role of STAT3 in Cancer

Although STAT3;s role as a tumor promoter has been widely demonstrated, several studies indicate that STAT3, under specific conditions, can act as a tumor suppressor factor in many different tumors (Table 1).

### 6.1. Glioblastomas

The first dual function of STAT3 was described in the nervous system during the development of cells along the glial lineage. The different cell types of the mammalian central nervous system (neurons, astrocytes, and oligodendrocytes) are derived from common neural precursor cells (NPCs) [117,118]. In the early stages of brain development, NPCs proliferate and generate neurons and glial cells in a sequential manner [119]. The differentiation of neural stem cells into astrocytes depends on the activation of two different factors: (i) the cytokine receptor leukemia inhibitory factor receptor β (LIFRβ—a receptor implicated in several cell differentiation processes) and (ii) STAT3 [120,121]. These factors induce the blocking of proliferative processes followed by cell differentiation. However, STAT3 is implicated also in the renewal capacity of NPCs, supporting the proliferation of these cells [122]. This dual role of STAT3 has raised questions about the relevance of STAT3 in glial tumorigenesis. In fact, STAT3 activation has been described in human gliomas [123]. At the same time, other authors have provided evidence of the absence of STAT3 activity in a large percentage of gliomas [124,125].

These opposite functions could be determined by the interaction of STAT3 with different signals in the oncogenic environment. For example, STAT3 has a tumor suppressive activity in glioma cells with an intact PTEN function and an oncogenic role in EGFRvIII-expressing gliomas and glioblastomas [126].

PTEN is an oncosuppressor protein, and STAT3 plays a key role in the PTEN pathway to suppress malignant transformation of astrocytes. In this pathway, IL8 plays a critical role. IL8 is not expressed in normal brain but is expressed in human glioblastoma tumors where it promotes proliferation and invasiveness. Gene-profiling analyses of glioblastoma cells have shown that the IL8 gene is directly repressed by STAT3 [126].

In cells with intact PTEN function, the protein kinase AKT is directly inhibited by PTEN. AKT is an inhibitor of FOXO3, a transcription factor involved in several biological functions including apoptosis. This transcription factor activates the transcription of the LIFRβ gene, which, in turn, activates STAT3, causing the repression of the IL8 gene, thereby inhibiting glioma cell proliferation and invasiveness. Conversely, in PTEN-deficient tumor cells, Akt is constitutively active, thus inhibiting FOXO3 function and leading to LIFRβ downregulation. In this scenario, STAT3 is not active and cannot repress the IL8 gene. This leads to an overregulation of IL8, which drives glioma cell proliferation and invasiveness [126].

In glioblastoma tumors that are not deficient in PTEN, STAT3 can play an oncogenic role in response to the expression of the oncogenic truncated protein EGFRvIII [29]. EGFRvIII forms a complex with STAT3 in the nucleus, converting the tumor-suppressive form of STAT3 into an oncogenic protein [126].

These findings indicate that STAT3 plays distinct roles in cell transformation depending on the mutational background of the tumor.

### 6.2. Prostate Cancer

Hyperactivation of the IL-6/STAT3 axis is frequently observed in prostate cancer cell lines. However, the blocking of the IL-6/STAT3 axis did not result in a survival advantage in patients with advanced prostate cancer. About 70% of metastatic prostate cancers show deletions or mutations in the PTEN gene. The loss of PTEN function in these tumors is associated with the activation of the oncosuppressor ARF–p53 pathway, which likely acts as a oncosuppressor compensatory mechanism [127]. In fact, in normal cells, ARF (p14ARF in humans; p19ARF in mice) is readily degraded, but it is stabilized in cells in which PTEN is deleted or mutated. ARF promotes the degradation of MDM2, a negative regulator of p53. Thus, loss of PTEN causes the activation of p53, which induces cycle arrest and apoptosis or senescence. It has been observed that genetic inactivation of STAT3 in a PTEN-deficient prostate cancer mouse model accelerates cancer progression by down-modulating ARF. In accordance with this experimental data, loss of STAT3 and ARF expression in patients with prostate cancer correlated with increased risk of disease recurrence and metastasis. ARF is a direct target of STAT3, which is required for the activation of the ARF-p53 pathway. Hence, STAT3 acts as an indirect tumor suppressor in prostate cancer, and the loss of STAT3 signaling disrupts the ARF–Mdm2–p53 tumor suppressor axis, promoting the progression of cancer [127].

### 6.3. Lung Cancer

The genetic background of tumor plays an important role in determining the activity of STAT3 as a pro-oncogenic or anti-oncogenic factor also in lung cancer. STAT3 is considered to play an oncogenic role in lung cancer [128,129]; consequently, STAT3 has been considered an important target for therapeutic intervention. The most frequent genetic alterations in lung adenocarcinomas are missense mutations and amplifications of the Kirsten rat sarcoma viral oncogene (KRAS) and EGFR. STAT3 is considered to play a tumor-promoting role in EGFR mutant non-small-cell lung cancer (NSCLC). In contrast, STAT3 plays an unexpected tumor suppressive role in KRAS mutant lung adenocarcinoma. This depends on the ability of STAT3 to control NF-κB-induced IL-8 expression by sequestering NF-κB within the cytoplasm, thereby inhibiting IL-8-mediated MDSCs and tumor vascularization, and hence tumor progression. Therefore, KRAS mutations in lung adenocarcinoma should be carefully considered in the therapeutic approach with STAT3 inhibitors [130].

### 6.4. Colorectal Cancer

Another case in which the oncosuppressor role of STAT3 is not dependent on its transcription function but the interaction with specific factors is colorectal cancer. The development of human colon adenomas is initially induced by mutations in tumor suppressor protein APC (adenomatous polyposis coli). APC is involved in the β-catenin/Wnt signaling pathway. The transition from adenoma to adenocarcinoma occurs only if two other oncosuppressor genes are mutated: SMAD4 (small mother against decapentaplegic) and EPHB3 (ephrin type-B receptor 3) genes. SMAD4 is a transcription factor that acts as a mediator of TGF-β signal transduction. When the structure of SMAD4 is altered, the expression of the genes involved in cell growth is deregulated, and cells proliferate without any inhibition [131]. EPHB3 is a protein implicated in several tumor-suppressive signaling pathways [132]. It has been observed that STAT3 knockdown causes in Apcmin/+ mice the transition from adenoma to carcinoma with the acquisition of an invasive phenotype. Therefore, in Apcmin/+ mice, STAT3 acts as a tumor suppressor similarly to SMAD4 and EPHB3. In this case, the tumor suppressor activity of STAT3 depends on its ability to suppress the expression of Snail-1, a zinc finger protein that acts as an inductor of the epithelial-mesenchymal transition. This is a process in which the epithelial tumor cells lose their epithelial phenotype and acquire a mesenchymal phenotype displaying reduced intercellular interactions and increased motility and invasiveness. Mechanistically, STAT3 facilitates glycogen synthase kinase (GSK) 3β-mediated degradation of SNAI by regulating the phosphorylation of GSK3β. [133].

### 6.5. Thyroid Cancer

STAT3 is a modulator of glycolysis and mitochondrial respiration, and constitutive activation of STAT3 induces aerobic glycolysis and downregulates mitochondrial activity both in primary fibroblasts and in STAT3-dependent tumor cell lines [134]. STAT3 induced aerobic glycolysis is dependent on the transcriptional and posttranscriptional induction of HIF1α. However, in thyroid cancer-derived cell lines, the absence of STAT3 led to a growth advantage under hypoxic stress because of a metabolic reprogramming characterized by increased glucose consumption, lactate production, and a reduced rate of oxygen consumption. Moreover, knockdown of STAT3 increases the expression of genes encoding glycolytic enzymes such as PDK1, GLUT3, and HK2. These effects seem to depend on an increased expression of HIF1α and its transcriptional targets, suggesting a paradoxical role in thyroid cancer for STAT3 as a negative regulator of HIF1α and aerobic glycolysis under hypoxic stress [135].

These data are consistent with the observation of high levels of activated phospho-STAT3 (Y705) in benign follicular thyroid adenomas and human primary papillary thyroid carcinoma without evidence of distant metastasis. Furthermore, a positive correlation between the expression of activated phospho-STAT3 (Y705) and smaller human primary papillary thyroid carcinoma tumor sizes has been observed. Finally, STAT3 knockdown resulted in the downregulation of multiple transcripts, including the tumor suppressor insulin-like growth factor binding protein 7 (IGFBP7) [135]. All these data strongly suggest a growth-suppressive role for STAT3 in human primary papillary thyroid carcinoma.

### 6.6. Breast Cancer

STAT3 is a regulator of epithelial cell death during mammary gland involution. Removal of the suckling stimulus and the consequent milk stasis lead to an increased expression of leukemia inhibitory factor (LIF), which is the initial activator of STAT3 during gland involution [136]. Activated STAT3 does not induce apoptosis in epithelial cells but activates a lysosome-mediated programmed cell death pathway (LM-PCD) by upregulating the expression of cathepsins B and L [137,138].

In contrast to this function, STAT3 acts as a promoter of survival in breast cancer cells [139,140]. In fact, several works have shown that STAT3 is constitutively active in invasive breast cancer biopsies, but not in biopsies from benign breast tumors, indicating that this transcription factor is involved in breast cancer progression [139,141]. Thus, STAT3 seems to have two opposite functions in normal epithelial cells of mammary gland and in breast cancer cells [140,142].

However, an analysis of 346 node-negative breast cancer showed that the expression of nuclear activated phospho-STAT3 (Y705) in the biopsied samples was correlated with a significant improved short-term (five years) and long-term (20-year) survival [143].

By studying the distribution of mutations in breast cancer metastases matched with their primary lesions, it was observed that mutations in synchronous metastases (mainly lymph nodal) were similar to the those of the primary breast tumor. These mutations typically interested genes frequently mutated in breast cancer, such as TP53, PIK3CA, and GATA3. In contrast, metachronous distant metastases had additional mutations, in particular mutations that caused loss of JAK2 and STAT3 functions [144]. This suggests that the JAK2/STAT3 system may act as a tumor suppressor in these metastases, explaining the improved short- and long-term survival in patients expressing nuclear activated phospho-STAT3 (Y705) in the biopsied samples. There are two possible explanations for this apparent paradox: (i) The JAK/STAT pathway’s inactivation could inhibit the immune response against metastases. This hypothesis is supported by the recent observation that JAK2 truncating mutations resulted in a lack of response to interferon gamma, including insensitivity to its antiproliferative effects on cancer cells [145]. (ii) JAK/STAT inactivation could induce cancer cells to exit from a quiescent state (G0/G1). In fact, under hypoxic conditions in the osteoblast niche, LIF receptor/STAT3 signaling confers a dormancy phenotype to disseminated breast cancer cells. Loss of the LIFR or STAT3 enables dormant breast cancer cells to downregulate dormancy-, quiescence-, and cancer stem cell-associated genes and to proliferate [146]. Moreover, STAT3 was identified as a dormancy-associated gene in estrogen receptor, ER-positive breast cancer cells [147].

### 6.7. Head and Neck Squamous Cell Cancers 

Constitutive activation of STAT3 has been described in head and neck squamous cell cancers (HNSCCs), where it is involved in deregulation of the cell cycle, increased cell growth, and inhibition of apoptosis [147,148,149,150]. In these cells, constitutive activation of STAT3 is dependent on TGF alpha-induced activation of the EGFR [150]. However, although the anti-apoptotic and cell growth inducing activity of STAT3 has been well documented, high nuclear STAT3 expression levels were associated with a favorable outcome in HNSCCs. Survival analysis in a cohort of 102 patients with HNSCCs showed that high nuclear STAT3 expression was associated with longer progression-free survival. Moreover, a multivariable analysis including different prognostic variables including gender, TNM stage, tumor grade, and tumor site showed that only STAT3 was significant, revealing a lower risk of progression and death for patients with high nuclear STAT3-expressing tumors. Since in the cohorts of this study, the majority of tumors were of advanced stage, it is possible to suppose that in an advanced stage, tumors become independent of STAT3-mediated pro-oncogenic signaling. In this case, STAT3 could function as a tumor suppressor as described in breast cancer or in glioblastoma.

### 6.8. STAT3 Isoforms

STAT3 has four isoforms: STAT3α, STAT3β, STAT3γ, and STAT3δ. STAT3α, STAT3β are generated by alternative splicing and differ structurally in their C-terminal transactivation domains because the β isoform lacks the C-terminal transactivation domain [151]. STAT3γ is derived from STAT3α by proteolysis during granulocytic differentiation. Like STAT β, it lacks the C-terminal transactivating portion. STAT3δ is an isoform expressed during the early stage of granulocytic differentiation.

STAT3α is the full-length version of STAT3. It regulates essential STAT3 target genes involved in cell proliferation, migration, and survival, and the oncogenic functions of STAT3 have also been associated with constitutively active STAT3α [152].

Conversely, STAT3β inhibits cancer progression acting as a repressor of STAT3. Several studies have shown that STAT3β can inhibit cell proliferation by impairing STAT3α-driven activation of Bcl-xL, p21, and cyclin D1 [153,154,155,156]. Moreover, STAT3β upregulates TRAIL receptor 2 and Fas expression favoring apoptosis in tumor cells [157,158]. STAT3β also plays a role in chemoresistance; in fact, administration of the STAT3β plasmid reduces chemoresistance and inhibits invasion of gastric cancer cells [159]. STAT3β has been shown to cause tumor regression in vivo [160,161], promote programmed cell death in myeloma cells [33], and impaired proliferation of melanoma, ovarian, and breast cancer cells [162,163,164]. STAT3β expression is an independent protective prognostic marker in patients with esophageal squamous cell carcinoma, which strongly correlated with longer overall survival and recurrence-free survival [165]. Therefore, the evaluation of both STAT3 isoforms in cancer might have an important role to understand the prognosis and the usefulness of a treatment with STAT3 inhibitors.

## 7. Treatment of Cancer with STAT3 Inhibitors

**Figure 3:**
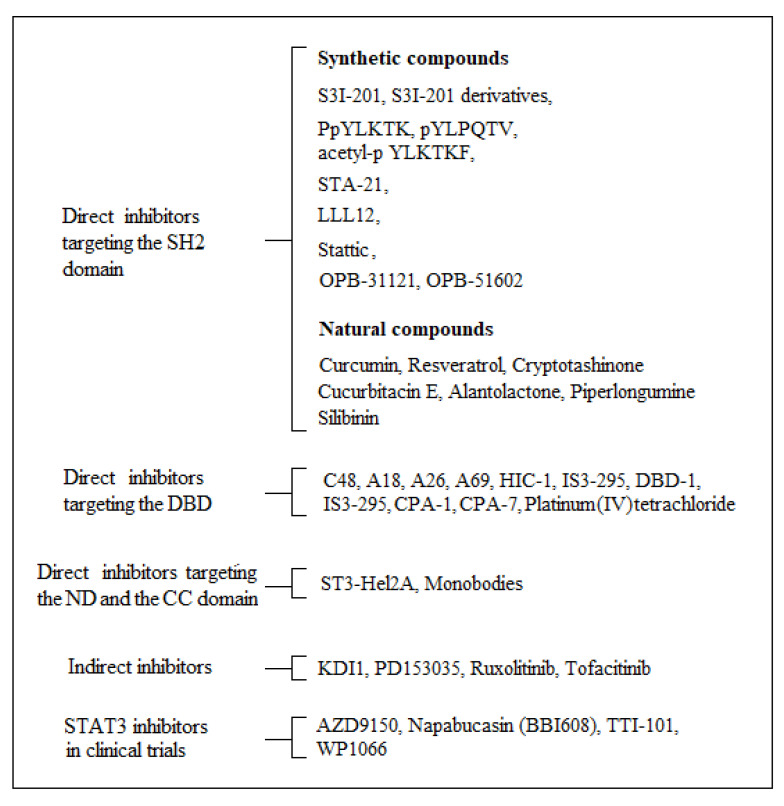
STAT3 inhibitors.

Since constitutive activation of STAT3 plays a central role in many tumors, small and non-small-molecular STAT3 inhibitors have been developed as novel cancer therapeutics [166,167,168]. Currently, there are two different classes of STAT3 inhibitor compounds: (i) direct inhibitors, which directly block different domains of STAT3; (ii) indirect inhibitors, which inhibit the signal transduction functions of STAT3, for example, by inhibiting the function of JAKs, Src, and Abl. The former inhibitors can be divided into four types according to the different STAT3 target domains (SH2, DBD, ND, and TAD). Figure 3 shows the most important STAT3 inhibitors targeting the STAT3 structural domains or the signal transduction functions of STAT3.

### 7.1. Inhibitors Targeting the SH2 Domain

SH2 domain interactions are critical for molecular activation and nuclear accumulation of phosphorylated STAT3 dimers to drive transcription. The SH2 domain mediates STAT3 dimerization via intermolecular phosphorylated tyrosine–SH2 interactions. Therefore, molecules able to block the SH2 domain of STAT3 have been considered for the treatment of different tumors. Many STAT3 inhibitors that block the SH2 domain were identified through a structure-activity relationship (SAR) study based on the previously identified inhibitor S3I-201. The key structural feature of these inhibitors is a salicylic acid moiety, which, by acting as a phosphotyrosine mimetic, facilitates binding to the STAT3 SH2 domain. S3I-201 and derivatives disrupt active STAT3:STAT3 dimers suppressing the expression levels of STAT3 target genes, such as Bcl-xL, survivin, cyclin D1, and MMP-9 and causing inhibition of cell growth, migration, and invasion of human cancer cells [169,170,171]. Other molecules able to block the SH2 domain of STAT3 include: (i) the STAT3 SH2 domain-binding peptides PpYLKTK [172], pYLPQTV [173], and acetyl-pYLKTKF [174]; (ii) STA-21 and its structurally optimized analog LLL12 [175]; (iii) stattic, a nonpeptidic small molecule [176].

OPB-31121 and OPB-51602 are two STAT3 inhibitors that interact with the SH2 domain with high affinity. Cell culture assays showed that OPB-31121 and OPB-51602 inhibited the phosphorylation of both Tyr705 and Ser727. OPB-31121 is a STAT3 inhibitor with potent antitumor effects against various human liver cancer cell lines [177]. In addition, OPB-31121 has shown the ability to decrease cell proliferation in both gastric cancer cells and in a xenograft model, induce apoptosis of gastric cancer cells, and inhibit the expression of antiapoptotic proteins [178]. Based on these findings, OPB-31121 has entered phase 1 and 2 clinical studies against hematopoietic and solid tumors, including hepatocellular carcinoma (HCC). However, in a phase 1 and pharmacological trial, OPB-31121 demonstrated insufficient antitumor activity for HCC and peripheral nervous system-related toxicities [179].

Another group of inhibitors targeting the SH2 domain of STAT3 are derivatives from natural compounds. Curcumin, a naturally-derived phytochemical from plants, has been shown to inhibit STAT3 phosphorylation and block the STAT3 signaling pathway in various tumor cell lines [180,181,182]. The stilbene compound resveratrol [183] and the natural compound cryptotanshinone, an extract from Salvia miltiorrhiza Bunge [184], are two other essential natural inhibitors of STAT3. Additionally, cucurbitacin E [185], alantolactone [186], piperlongumine [187], and silibinin [188] are natural STAT3 inhibitors that interact with the SH2 domain.

### 7.2. Inhibitors Targeting the DBD

DBD interacts with the promoter of STAT3 targeting genes and has a relatively high specificity. The small molecule C48 was the first inhibitor targeting the DBD. It acts by alkylating glutathione sulfhydryl on cysteine (C468) in the STAT3 DBD region. C48 blocks the accumulation of activated STAT3 in the nucleus in tumor cell lines that overexpress active STAT3, leading to impressive inhibition of tumor growth in mouse models [189]. Using an in silico screening approach, three STAT3 inhibitor, denominated A18, A26, and A69, targeting the DBD of STAT3, were identified. These compounds were found to inhibit STAT3-specific DNA binding activity, suppress the proliferation of cancer cells harboring aberrant STAT3 signaling, inhibit the migration and invasion of cancer cells, and inhibit the STAT3-dependent expression of downstream targets by blocking the binding of STAT3 to the promoter regions of responsive genes in cells. In addition, A18 was able to reduce tumor growth in a mouse xenograft model of lung cancer with little effect on body weight [190]. Other compounds targeting the DBD of STAT3 include HIC-1 [191] and DBD-1 [192].

Finally, several platinum compounds, including IS3-295, CPA-1, CPA-7, and platinum (IV) tetrachloride, have shown the ability to inhibit the STAT3 DBD, suggesting potential new applications for platinum complexes as modulators of the STAT3 pathway for cancer therapy [193,194].

### 7.3. Inhibitors Targeting the ND and the CC Domain

The N-terminal domain plays an important role in the interaction of dimers of STAT-3 and in the attachment of STAT-3 dimers to the sites of DNA. Therefore, in the last few years, compounds able to interact and block this domain have been developed. ST3-H2A2 is a highly selective STAT3 ND inhibitor. This compound induces apoptotic death in cancer cells associated with potent activation of proapoptotic genes. In particular, it activates the expression of the proapoptotic gene CHOP, causing apoptosis in tumor cells [195]. ST3-Hel2A is a synthetic peptide that selectively targets the N-terminal domain of STAT-3 and inhibits dimerization. In preclinical trials, ST3-Hel2A was found to inhibit the STAT-3 N-terminal domain, resulting in the blockage of STAT-3 dimerization and apoptosis in prostate cancer cell lines.

Synthetic antibody mimetics, termed monobodies, have been developed to interfere with STAT3 N-terminal and coiled-coil domain signaling. These monobodies are highly selective for STAT3 and bind to STAT3 with nanomolar affinity. The crystal structure of STAT3 in complex with monobody MS3-6 reveals bending of the coiled-coil domain, resulting in diminished DNA binding and nuclear translocation. MS3-6 expression strongly inhibits STAT3-dependent transcriptional activation and disrupts STAT3 interaction with the IL-22 receptor [196].

### 7.4. Indirect Inhibitors

STAT3 indirect inhibitors prevent STAT3 activation by inhibiting the tyrosine kinase events at the receptor level. Receptor-associated and non-receptor tyrosine kinases are critical upstream regulators of STAT3 activation, so targeting these kinases has attractive potential to block STAT3 activation. Molecules that indirectly target upstream activators of STAT3 include KDI1, a short peptide that specifically binds to the intracellular domain of the EGFR [197], PD153035, an RTK inhibitor [198], and several JAK and Src kinase inhibitors [199,200,201,202,203,204,205].

Numerous JAK2 inhibitors have been developed that are able to inhibit the JAK2/STAT signaling pathways, and ruxolitinib and tofacitinib have been approved by the FDA for the treatment of myelofibrosis and rheumatoid arthritis. However, apart from myelofibrosis, no satisfactory results have been obtained with JAK2 inhibitors in cancer patients, and clinical trials with JAK1/2 inhibitors and Src inhibitors have shown limited efficacy or excessive toxicity in advanced solid tumors [206].

### 7.5. Clinical Trials

Despite the large amount of STAT3 inhibitors studied in vitro, only a few compounds have entered clinical trials. AZD9150, napabucasin (BBI608), TTI-101, and WP1066 are the most studied compounds in different trials.

AZD9150 is a 16 oligonucleotide antisense molecule (ASO) targeting the 3’ untranslated part of STAT3 [207]. Clinical trials with AZD9150 in patients with advanced/metastatic carcinoma have shown the safety and anti-tumor activity of this compound. When used in combination with durvalumab, AZD9150 seems able to control advanced pancreatic, lung, and colorectal cancer.

Napabucasin (BBI608) is a newly-developed small molecule inhibitor of STAT3 [208] currently studied in several clinical trials in patients with metastatic pancreatic adenocarcinoma, colorectal cancer, gastric cancer, glioblastoma, hematologic malignancy, malignant pleural mesothelioma, and hepatocellular carcinoma.

TTI-101 is an orally bioavailable, binaphthol-sulfonamide-based inhibitor of STAT3 that specifically targets and binds to the phosphotyrosyl peptide binding site within the SH2 domain of STAT3 [209]. It is in a phase I trial in advanced cancers.

WP1066 has been designed to target the STAT3 pathway in tumor cells [210], and phase I trial studies have been started in patients with recurrent, or refractory, or progressive malignant brain tumors.

## 8. Conclusions

STAT3 is a transcription factor that has been extensively studied over the past 20 years due to its implication in many biological processes and diseases, including cancer. It is one of the most complex transcription regulators involved in multiple cellular processes, and its hyperactivation has been reported in many types of tumors. This has led to this transcription factor being considered an important target for the development of a new class of drugs. Despite a large number of STAT3 inhibitors having been developed and studied in vitro, only a few of them are currently in clinical trials, and at present, there are no clinically approved drugs directly targeting STAT3. This is, in part, caused by the serious toxic effects observed with the use of most STAT3 inhibitors. In fact, STAT3 is involved in many biological processes, and its inhibition can induce important alterations in normal cells. Furthermore, complete inhibition of the STAT3 signaling pathway may require very high drug concentrations as compared to inhibitors of ligands or cell surface receptors. In fact, cellular signaling transmitted by the latter is always amplified downstream, while STAT3 translates the signal without amplification. Finally, several studies have raised questions and doubts about the use of STAT3 inhibitors based on the tumor suppressor role of STAT3 in several cancers. This role does not depend on the transcription function of STAT3 but on its ability to interact with other biological factors. Therefore, the goal could be the discovery of high-quality targeted selective agents against STAT3 that do not affect the functions of healthy cells and the identification of patients who are likely to receive actual benefit from the treatment.

## Figures and Tables

**Table 1:** Tumor suppressor and tumor promoter mechanisms of Signal transducer and activator of transcription 3 (STAT3) in different malignancies.

Tumor	Tumor Background	Tumor Suppressor Activity	Tumor Promoter Activity
Glioma	Normal PTEN expression	LIFRβ activation of STAT3 that inhibits IL-8 expression.	
	EGFRvIII expression		Formation of oncogenic EGFRvIII-STAT3 complex.
Prostate cancer		STAT3 activation of ARF-p53 pathway.	
Lung Adenocarcinomas	KRAS mutations	STAT3 dependent sequestration of NF-kB in the cytoplasm and inhibition of IL-8 expression.	
	EGFR mutations		STAT3 tumor promoter activity.
Colorectal cancer		STAT3 mediated degradation of Snail-1 and inhibition of epithelial-mesenchymal transition.	STAT3 mutations associated with APC mutations.
		STAT3β expression.	
Thyroid cancer		STAT3 dependent suppression of HIF1α and aerobic glycolysis under hypoxic stress.	
		STAT3 dependent activation of IGFBP7.	
Breast cancer	Primary tumor		STAT3 tumor promoter activity.
	Metastasis	STAT3 activation of dormancy-associated genes under hypoxic condition.	
Esophageal squamous cell carcinoma			STAT3α expression.
Esophageal squamous cell carcinoma with longer overall survival		STAT3β expression.	
